# Chromosome-level genome assembly and annotation of *Clanis bilineata tsingtauica* Mell (Lepidoptera: Sphingidae)

**DOI:** 10.1038/s41597-024-03853-5

**Published:** 2024-09-30

**Authors:** Yulu Yan, Ke Zhao, Longwei Yang, Nan Liu, Yufei Xu, Junyi Gai, Guangnan Xing

**Affiliations:** Laboratory of Biology and Genetics Improvement of Soybean, Ministry of Agriculture, Zhongshan Biological Breeding Laboratory (ZSBBL), National Innovation Platform for Soybean Breeding and Industry Education Integration, State Key Laboratory of Crop Genetics & Germplasm Enhancement and Utilization, Jiangsu Collaborative Innovation Center for Modern Crop Production, College of Agriculture, Nanjing Agricultural University, Nanjing, 210095 China

**Keywords:** Open reading frames, Agricultural genetics, Eukaryote, Comparative genomics

## Abstract

The soybean hawkmoth *Clanis bilineata tsingtauica* Mell (Lepidoptera, Sphingidae; CBT), as one of the main leaf-chewing pests of soybeans, has gained popularity as an edible insect in China recently due to its high nutritional value. However, high-quality genome of CBT remains unclear, which greatly limits further research. In the present study, we assembled a high-quality chromosome-level genome of CBT using PacBio HiFi reads and Hi-C technologies for the first time. The size of the assembled genome is 477.45 Mb with a contig N50 length of 17.43 Mb. After Hi-C scaffolding, the contigs were anchored to 29 chromosomes with a mapping rate of 99.61%. Benchmarking Universal Single-Copy Orthologues (BUSCO) completeness value is 99.49%. The genome contains 252.16 Mb of repeat elements and 14,214 protein-coding genes. In addition, chromosomal synteny analysis showed that the genome of CBT has a strong synteny with that of *Manduca sexta*. In conclusion, this high-quality genome provides an important resource for future studies of CBT and contributes to the development of integrated pest management strategies.

## Background & Summary

**Fig. 1:**
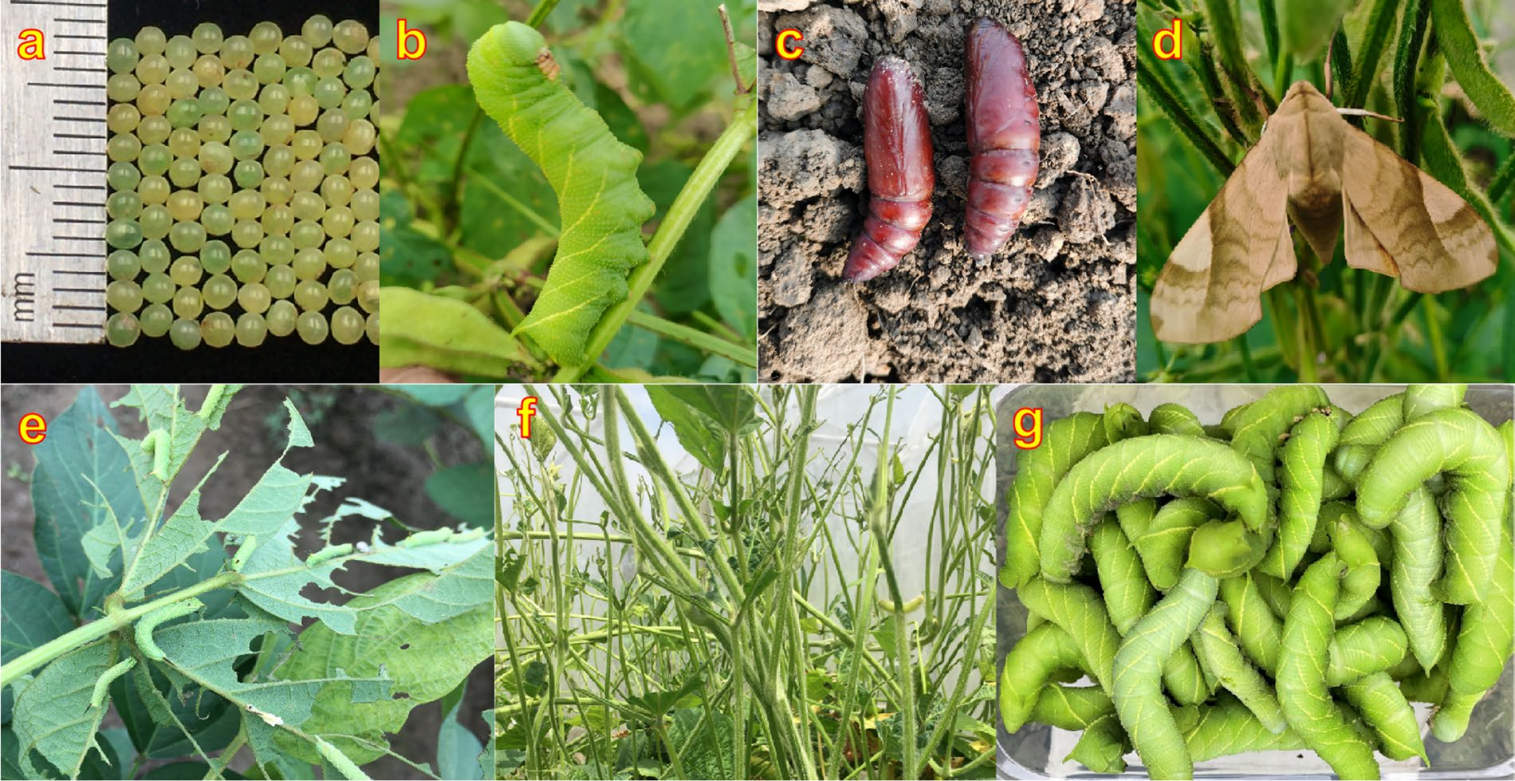
Developmental stages of CBT and its damage to soybeans. (**a**) Egg. (**b**) Fifth instar larva. (**c**) Pupa. (**d**) Adult. (**e**) Damaged soybean leaves and low instar larvae on them. (**f**) Damaged soybean by fifth instar larvae in net room. (**g**) Harvested fifth instar larvae from artificial rearing.

The soybean hawkmoth *Clanis bilineata tsingtauica* Mell, (Lepidoptera, Sphingidae, Clanis; CBT), an agricultural pest infesting soybean, is mainly distributed in China, Japan, and the Korean Peninsula^1^. The CBT larvae has five instars, and the fifth instar is the larval gluttonous stage^2^. In severe cases, the larvae can lead to only the stems remain of the plant, crop failure, or even plant death^3^ (Fig. 1).

Meanwhile, CBT has a long history of consumption as a crucial edible insect in China^4^. The 5th instar larval meat is used freeze-dried, fried, soup and canned^5^. The larvae of CBT are nutrient-rich and have abundant essential amino acids, which can be used as a high-quality protein source^6^. At present, CBT is mainly obtained through artificial rearing^7^. The artificial rearing of CBT has become a promising agricultural industry in China, with an annual production of 30,000 tons and an output value of nearly 620 million dollars^8^.

Sphingidae has about 1,500 insects worldwide^9^, and many of which are considered significant agricultural pests, such as the tobacco hornworm (*Manduca sexta*) and sweet potato hornworm (*Agrius convolvuli*). However, the genome of hawkmoth has been poorly studied. To date, genome assembly can been retrieved for only 14 species of Sphingidae (as of January 2024 from NCBI), including *Hyles lineata* (Macroglossinae)^10^, *Hyles euphorbiae* (Macroglossinae)^11^, *Mimas tiliae* (Smerinthinae)^12^, *Deilephila porcellus* (Macroglossinae)^13^, and *M. sexta* (Sphinginae)^14^.

In the present study, we assembled a chromosome-level genome of CBT for the first time using PacBio HiFi reads and Hi-C sequencing technologies. We annotated repeat elements, non-coding RNAs (ncRNAs), and protein-coding genes of this genome. Additionally, we performed chromosomal synteny analysis of the CBT genome with those of *Bombyx mori* and *M. sexta*. The high-quality genome of CBT is greatly helpful for understanding and conducting further study of utilization as edible insect, damage mechanism and relevant integrated pest management strategies of sphingid species.

## Methods

### Sample collection and sequencing

The sample of fifth instar CBT larvae were collected from soybean field, and its original population derived from Lianyungang, Jiangsu Province, China. Subsequently, larvae were placed in incubator with a temperature of 26 ± 1°C, relative humidity of 60% ± 10%, and photoperiod of 14 h L: 10 h D. After two days of starvation treatment, washed the larvae with distilled water and placed them in liquid nitrogen.

Genomic DNA from CBT was extracted using the CTAB method. According to the manufacturer’s instructions, a short-read library was constructed using the Agencourt AMPure XP-Medium kit with an insert size of 200‒400 bp and was sequenced on DNBSEQ-T7 platform. A PacBio HiFi library with an insert size of 15 Kb was constructed using the SMRTbell® Express Template Prep Kit 2.0. And HiFi library was sequenced on PacBio Sequel IIe platform. The Hi-C sequencing was carried out by digesting extracted DNA with the Mbol restriction enzyme on Illumina Xplus platform. Next-generation RNA-seq library was constructed using the VAHTS mRNA-seq v2 Library Prep Kit and also was sequenced on Illumina Xplus platform. The third-generation full-length RNA sequencing library of Oxford Nanopore Technologies (ONT) was constructed using the SQK-PCS109 + SQKPBK004 Kit by BenaGen (Wuhan, China) and sequenced on Oxford Nanopore PromethION platform. All library constructions and sequencing were completed by Berry Genomic (Beijing, China), expect the construction and sequencing of ONT RNA library. Finally, we obtained 30.59 Gb (64.07×) of Whole-Genome Sequencing (WGS) raw data, 36.70 Gb (76.87×) of HiFi data, 74.68 Gb (156.42×) of Hi-C data, 11.81 Gb of RNA-seq data, and 13.71 Gb of RNA-ONT data (Table 1) with high quality (Tables S1–S5).

**Table 1:** Statistics of sequencing data of *C. bilineata tsingtauica*.

Library	Reads	Raw data (Gb)	Average length (bp)	Coverage (×)
WGS	203,937,730	30.59	150	64.07
HiFi	2,278,055	36.70	16,110.4	76.87
Hi-C	497,879,266	74.68	150	156.42
RNA-seq	78,703,000	11.81	150	—
RNA-ONT	11,252,664	13.71	1,218.35	—

### Genome assembly

**Fig. 2:**
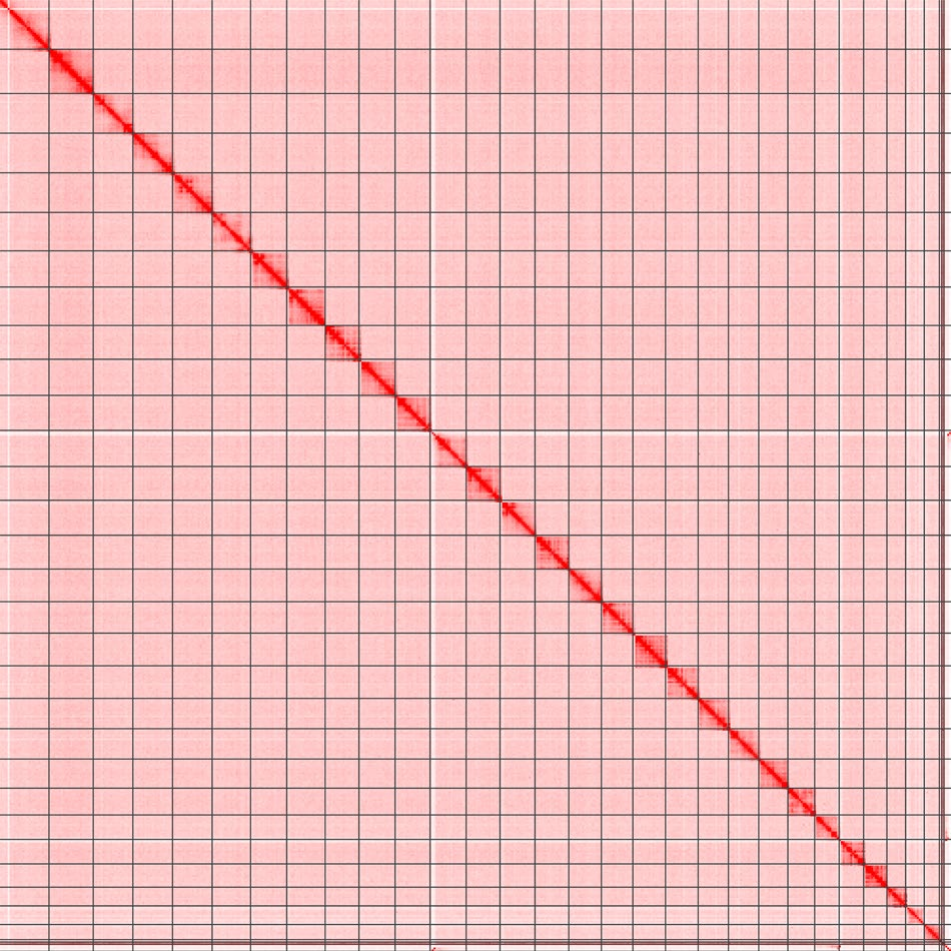
Hi-C interaction heatmap of *C. bilineata tsingtauica*.

We used pbccs v6.4.0 (https://github.com/PacificBiosciences/ccs) to filter low-quality HiFi reads below Q20 base quality. Then we used Hifiasm v0.19.6^15^ with default parameters for the initial round of assembly and only retained contig assembly sequences with coverage depth exceeding 6×. Subsequently, Hi-C data and the YAHS v1.2^16^ pipeline were utilized for anchoring contigs onto chromosomes and assembly. Hi-C data was quality controlled and aligned to the genome using chromap v0.2.5^17^. Two rounds of scaffolding were performed using YAHS v1.2 with default parameters. The assembly results from the initial round of scaffolding were manually corrected using Juicebox v1.11.08^18^, then performed the second round of scaffolding. The sequencing coverage of each pseudochromosome was evaluated by SAMtools v1.108 (https://www.htslib.org). The Hi-C interaction heatmap reveals a remarkably high quality of scaffolding (Fig. 2). We used MMseq 2 v13^19^ to perform blastn-like searches to detect potential contaminants in the assembly based on the NCBI nt and UniVec databases. Minimap2 (https://github.com/lh3/minimap2) was used to align reads back to the genome assembly. Compleasm v0.2.4^20^ based on insecta_odb10 dataset (n = 1,367 orthologues) and merqury v1.3^21^ were respectively used to assess completeness of Benchmarking Universal Single-Copy Orthologues (BUSCO) and the single-base quality value (QV).

Finally, we obtained the high-quality chromosome-level genome of CBT, with the genome size of 477.45 Mb and GC content of 38.55% (Table 2). The assembly included 66 contigs and 56 scaffolds, with both scaffold N50 and contig N50 lengths of 17.43 Mb. 475.61 Mb of contigs were anchored to 29 pseudochromosomes, with a rate of 99.61%. The BUSCO assessment of genome completeness was 99.49% (C), with only 0.15% duplicated BUSCOs (D), 0.15% fragmented BUSCOs (F), and 0.37% missing BUSCOs (M). The mapping rates for WGS, HiFi, RNA-seq, and RNA-ONT data were 97.86%, 99.90%, 95.78%, and 90.39%, respectively (Table 2). Chromosome 29 was the shortest, with a length of 8,386,962 bp, while chromosome 22 was the longest at 25,470,929 bp. The overall average length of the chromosomes was 16,400,209 bp. In terms of sequencing quality, the mean QV across all chromosomes was approximately 58, while the average sequencing coverage depth was about 70× for HiFi and 61× for WGS (Table 3). These indicators suggest that the assembly of CBT genome is of extremely high quality in terms of completeness and continuity. In addition, we found a complete mitochondrial whole genome sequence in the genome assembly, with a length of 15,417 bp and annotated by MitoZ v3.6^22^ (Fig. S1).

**Table 2:** The chromosomal-level genome assembly statistics of *C. bilineata tsingtauica*.

Genome assembly	Results
Genome size (Mb)	477.45
Number of pseudo-chromosomes	29
Anchored to chromosome (Mb, %)	475.61 (99.61%)
Number of contigs	66
Contig N50 (Mb)	17.43
Longest contig length (Mb)	21.648
Number of scaffolds	56
Scaffold N50 (Mb)	17.43
Longest scaffold length (Mb)	25.471
GC content (%)	38.55
BUSCO completeness (C, %)	99.49
Complete and single-copy BUSCOs (S)	99.34
Complete and duplicated BUSCOs (D)	0.15
Fragmented BUSCOs (F)	0.15
Missing BUSCOs (M)	0.37
Mapping ratio of reads (%)	
WGS	97.86
HiFi	99.90
RNA-seq	95.78
RNA-ONT	90.39

**Table 3:** Genome assembly summary of length, sequencing coverage and QV value for each chromosome.

Pseudochromosome	Length (bp)	HiFi (×)	WGS (×)	QV
Chr01	21,842,936	75.9498	63.5229	56.7555
Chr02	8,992,602	64.9312	62.6694	55.3017
Chr03	16,494,334	71.3470	61.2169	63.5307
Chr04	20,181,594	72.3723	61.8389	59.6149
Chr05	20,027,834	74.0953	62.0599	58.1770
Chr06	17,762,429	72.4008	61.2941	56.7883
Chr07	14,163,584	70.0379	60.2654	58.3886
Chr08	17,196,339	70.7947	61.0319	58.8274
Chr09	18,094,634	72.9921	61.8105	59.0283
Chr10	19,607,446	72.8832	61.7446	57.7114
Chr11	17,930,107	72.4073	61.4835	57.2412
Chr12	18,506,232	71.7772	61.4748	54.2548
Chr13	18,502,477	73.1665	61.4931	59.1352
Chr14	14,131,661	68.4996	60.3523	59.0780
Chr15	20,258,944	72.4246	61.6154	57.7783
Chr16	15,130,695	69.3817	60.6863	59.5346
Chr17	18,065,600	71.1292	60.8846	61.0263
Chr18	16,792,605	72.2128	61.0523	56.4834
Chr19	15,667,469	70.3254	61.1012	59.4483
Chr20	12,493,053	65.5274	59.3905	52.3332
Chr21	16,266,667	71.3860	60.9854	58.7939
Chr22	25,470,929	73.4463	62.3269	58.4343
Chr23	17,430,162	72.0736	61.3113	57.3973
Chr24	10,189,500	63.9654	59.2680	58.8896
Chr25	15,981,465	70.5920	60.7040	58.4789
Chr26	16,763,654	72.0847	61.5593	59.0323
Chr27	12,103,761	67.6083	59.7874	60.8292
Chr28	11,170,395	64.7877	59.6851	57.8649
Chr29	8,386,962	58.6350	57.5730	55.3117
Average	16,400,209	70.3185	61.0410	58.1196

The naming of chromosomes is based on homology with the chromosomes of *B. mori*.

### Genome annotation

**Fig. 3:**
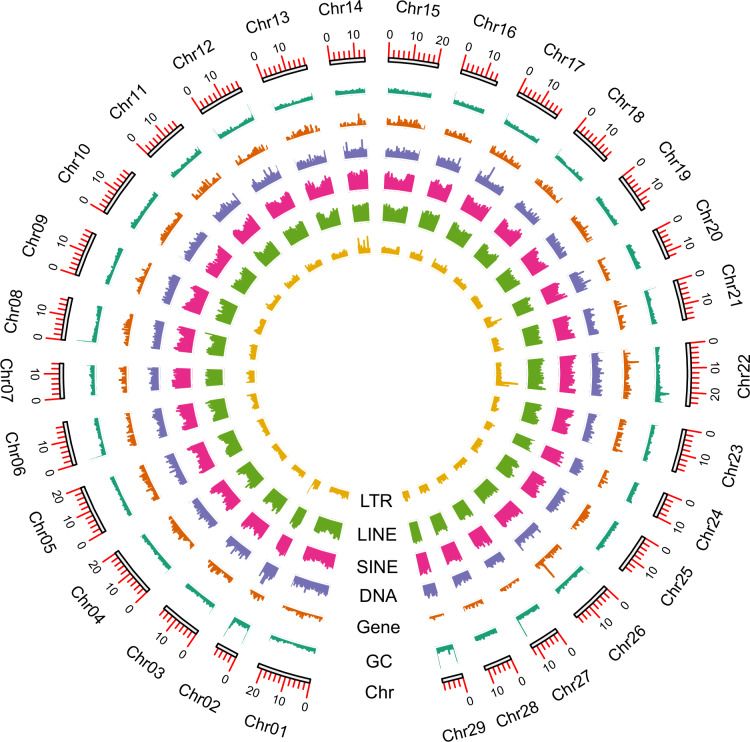
Genome characteristics of *C. bilineata tsingtauica* (window size 100 kb). From the outer ring to the inner ring are the distributions of chromosome length, GC content, gene density, TE (DNA, SINE, LINE, and LTR).

We employed RepeatModeler v2.0.5^23^ and the “LTRStruct” LTR discovery pipeline to construct a repeat library. This library was combined with the Dfam 3.7^24^ and RepBase-20181026^25^ databases to form a custom library. Repeat elements were identified by aligning the genome with the custom library using RepeatMasker v4.1.5^26^. The analysis revealed 252.16 Mb repeat elements, accounting for 52.81% of the genome. The major repeat elements included LINEs (14.73%), SINEs (14.56%), Unclassified (12.43%), LTRs (3.60%), Rolling-circles (3.42%), and DNA elements (3.06%) (Table 4; Table S6). Subsequently, Infernal v1.1.5^27^ searched for non-coding RNAs based on Rfam database. And tRNAs were predicted using tRNAscan-SE v2.0.12^28^. Low-confidence tRNAs were filtered using the built-in ‘EukHighConfidenceFilter’ script. In total, we annotated 1,434 ncRNAs, mainly including 170 rRNAs, 74 miRNAs, 76 snRNAs, and 636 tRNAs (Table 4; Table S7). Moreover, genome characteristic visualization was performed with TBTools-II v2.042^29^ in combination with annotation (Fig. 3).

**Table 4:** Genome annotation statistics of *C. bilineata tsingtauica*.

Annotation category	Results
Repeat elements	
LINEs (Mb)	70.32 (14.73%)
SINEs (Mb)	69.52 (14.56%)
Unclassified (Mb)	59.35 (12.43%)
LTRs (Mb)	17.20 (3.60%)
Rolling-circles (Mb)	16.32 (3.42%)
DNA elements (Mb)	14.61 (3.06%)
Total	252.16 (52.81%)
Non-coding RNAs	
Number of rRNA	170
Number of miRNA	74
Number of snRNA	76
Number of tRNA	636
Number of ribozyme	3
Number of lncRNA	3
Total	1,434
Protein-coding genes	
Number of protein-coding genes	14,214
Mean protein length (aa)	642
Mean gene length (bp)	16,966.9
Number of exons per gene	7.7
Mean exon length (bp)	314.6
Number of introns per gene	6.7
Mean introns length (bp)	2,347.9
Number of CDSs per gene	7.4
Mean CDS length (bp)	222.5
Number of genes matching Uniprot records	13,889
Number of genes with InterProScan annotations	11,694
Number of genes with GO items	10,190
Number of genes with KEGG pathways items	4,863

Number of genes with GO items and KEGG pathways items are the results combining InterProScan and eggNOG.

Protein-coding genes were annotated using MAKER v3.01.04^30^ by integrating three strategies: *ab initio* prediction, transcriptome-based and homology-based prediction. BRAKER v3.0.6^31^ and GeMoMa v1.9^32^ were used to integrate transcriptome and protein evidence, with their prediction results combined as *ab initio* input file for MAKER. Transcriptome alignment BAM files were generated using HISAT2 v2.2.1^33^. BRAKER automatically trained Augustus v3.4.0^34^ and GeneMark-ETP^35^, and combined transcriptome data and arthropod homologous protein sequences from OrthoDB11 database^36^ to improve prediction accuracy. Additionally, homology-based prediction was performed using GeMoMa based on the annotation of genes of *Drosophila melanogaster* (Diptera), *M. sexta* (Lepidoptera), *Amyelois transitella* (Lepidoptera), *B. mori* (Lepidoptera), and *Spodoptera frugiperda* (Lepidoptera) from GenBank (Table 5). For transcriptome-based prediction approach, the transcriptome was assembled using StringTie v2.2.1^37^, and BAM files were generated with HISAT2.

**Table 5:** Genome datasets were used for gene prediction based on homology in the study.

Species	Order	Family	Source
*Drosophila melanogaster*	Diptera	Drosophilidae	NCBI(GCF_000001215.4)
*Amyelois transitella*	Lepidoptera	Pyralidae	NCBI(GCF_032362555.1)
*Spodoptera frugiperda*	Lepidoptera	Noctuidae	NCBI(GCF_023101765.2)
*Clanis bilineata tsingtauic*	Lepidoptera	Sphingidae	this study(GCA_036417725.1)
*Manduca sexta*	Lepidoptera	Sphingidae	NCBI(GCF_014839805.1)
*Bombyx mori*	Lepidoptera	Bombycidae	NCBI(GCF_014905235.1)

In the end, we predicted 14,214 protein-coding genes in the CBT genome by using MAKER, with an average gene length of 16,966.9 bp. The average number of exons, introns, and CDS of each gene were 7.7, 6.7, and 7.4, respectively (Table 4). The average length of exons, introns, and CDS of each gene were 314.6 bp, 2,347.9 bp, and 222.5 bp, respectively (Table 4). What’s more, BUSCO completeness of the predicted protein-coding gene sequences was 98.90%, including 77.47% single-copy, 21.43% duplicated, 0.07% fragmented, and 1.02% missing BUSCOs.

Functional annotation of the genes was performed using Diamond v2.1.7.161^38^ (-very-sensitive -e 1e-5) by searching against the UniProtKB v202305 database. For further gene functional annotation, InterPro 5.65–97.0^39^ was used to search databases including Pfam^40^, SMART^41^, Superfamily^42^, and CDD^43^. The eggNOG v5.0.2^44^ database (http://eggnog6.embl.de) was searched by eggNOG-mapper v2.1.12^45^. After integrating these results, we found that 13,889 (97.71%) genes were functionally annotated against the UniProtKB database. InterPro identified structural domains for 11,694 protein-coding genes. InterPro and eggNOG-mapper jointly annotated GO terms for 10,190 genes and KEGG pathways for 4,863 genes (Table 4)

### Chromosomal synteny analysis

**Fig. 4:**
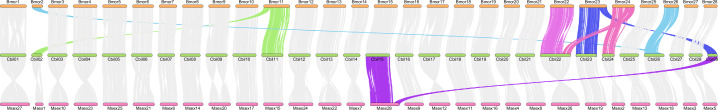
Chromosomal synteny among *C. bilineata tsingtauica* (Cbil), *Bombyx mori* (Bmor) and *Manduca sexta* (Msex). Color stripes represent the major occurrence of chromosomal fissions or fusions.

In order to explore interspecific chromosomal relationships, chromosomal synteny analysis was conducted for CBT compared with *B. mori* (Lepidoptera) and *M. sexta* (Lepidoptera) (Table 5). Protein sequences were aligned using Diamond with parameter of “--ultra-sensitive --iterate -e 1e-5 -k 5”. Subsequently, chromosomal synteny was analyzed using MCScanX^46^ with the parameter of “-s 5 -e 1e-5”. The results indicated that exceedingly notable synteny between the genome chromosomes of CBT and *M. sexta* was observed (Fig. 4). A chromosomal fission or fusion events occurred between *M. sexta* Chr28 and CBT Chr15 + Chr29. The synteny between the chromosomes of CBT and *B. mori* genome was also strong but slightly lower than that between CBT and *M. sexta*, and chromosomal fusion or fission events were more frequent. Moreover, the autosomes and sex chromosome Z were also determined by chromosome synteny, according to the relatively conserved feature in the Lepidoptera sexual chromosome Z^47^. Conclusively, the chromosome 1 was confirmed Z chromosome by sharing high synteny features with *B. mori* and *M. sexta* Z chromosomes (Fig. 4).

## Data Records

The Hi-C, PacBio HiFi, ONT RNA seq, RNA seq, and WGS data for the CBT genome can be found on NCBI with the accession numbers SRR27748981‒SRR27748985^48^ under BioProject accession number PRJNA1060222^49^. The assembled genome has been deposited in the NCBI assembly with the accession number GCA_036417725.1^50^. Additionally, the annotation results of the CBT genome have been stored in the Figshare^51^.

## Technical Validation

Three methods were used to assess the quality of the CBT genome assembly. Firstly, the purity of the genome DNA was verified using a NanoDrop 2000 spectrophotometer and Qubit fluorometric quantitation. The integrity of the genome DNA was checked via pulsed-field gel electrophoresis and agarose gel electrophoresis. The absorbance at 260/280 nm was approximately 1.89. Secondly, we used compleasm v0.2.4 with the insecta_odb10 database (n = 1,367 orthologues) as a reference to assess the completeness of the genome assembly. The assessment showed that the completeness of BUSCO was 99.49%, including 99.34% single-copy BUSCOs, 0.15% duplicated BUSCOs, 0.15% fragmented BUSCOs, and 0.37% missing BUSCOs (Table 2). The predicted protein-coding gene sequences were evaluated for BUSCO completeness, resulting in C: 98.90% [S:77.47%, D:21.43%], F:0.07%, M:1.02%. Thirdly, reads were aligned back to the assembly results using Minimap2, and the mapping rates for WGS, RNA-seq, RNA-ONT, and HiFi data were all over 90% (Table 2).

## Supplementary information

Supplementary Tables

Fig. S1 The mitochondrial whole genome sequence of <Emphasis Type="italic">C. bilineata</Emphasis> tsingtauica.

## Data Availability

No specific script was used in the present study. All commands and pipelines used of this work in data processing were performed according to the manual and protocols of the relevant bioinformatic software. All commands used in this work could be inquired in the Figshare^52^.
