# Supplementary figures and images for: Chromosome-level genome assembly and annotation of Clanis bilineata tsingtauica Mell (Lepidoptera: Sphingidae)
Source: Sci Data. 2024 Sep 30;11:1062. doi: 10.1038/s41597-024-03853-5 (PMC11443141; doi:10.1038/s41597-024-03853-5)

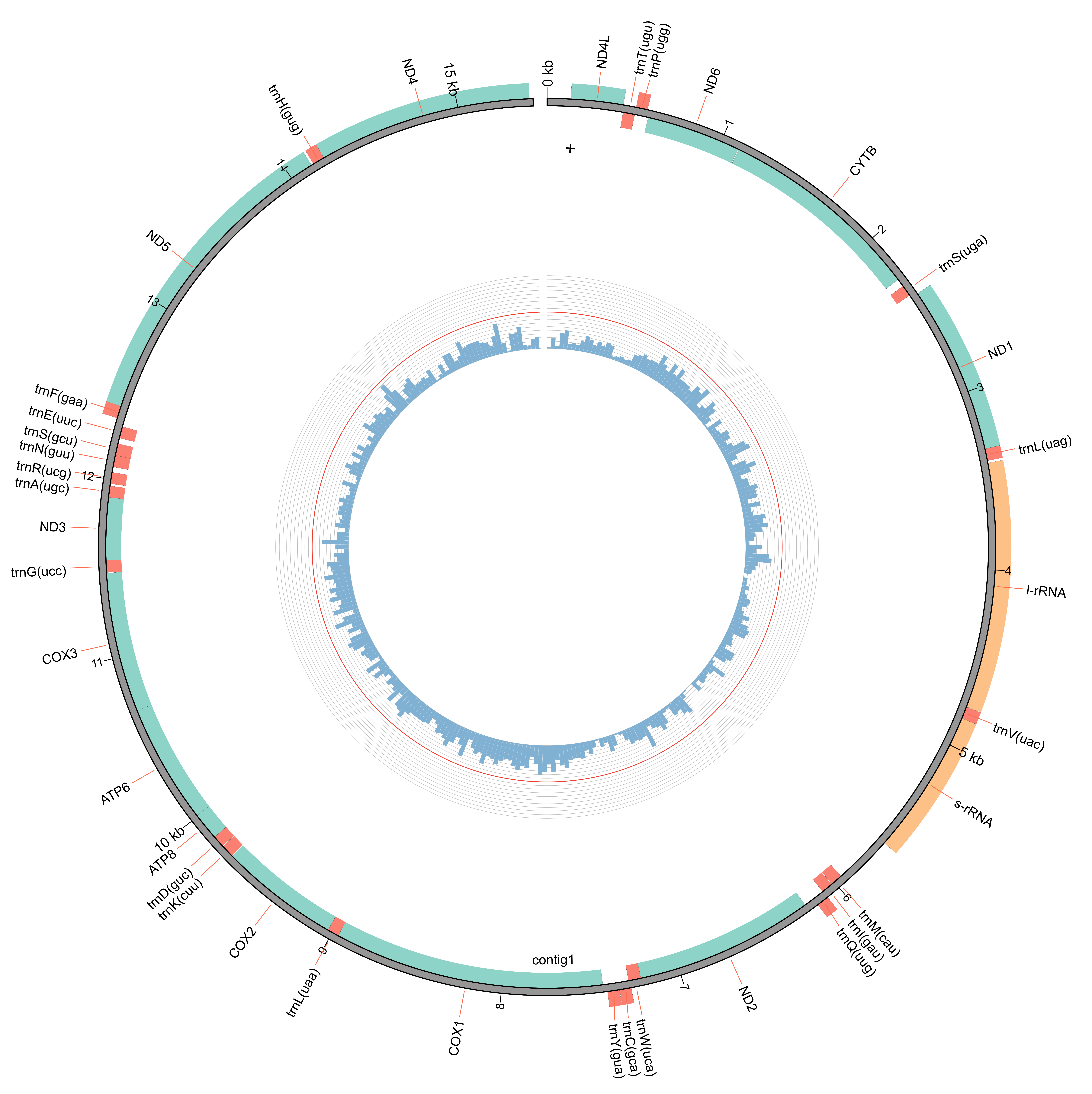

Supplement: Supplementary file 2 — Fig. S1 The mitochondrial whole genome sequence of C. bilineata tsingtauica. [file 41597_2024_3853_MOESM2_ESM.pdf]
